# The shaping of human diversity: filters, boundaries and transitions

**DOI:** 10.1098/rstb.2015.0241

**Published:** 2016-07-05

**Authors:** Marta Mirazón Lahr

**Affiliations:** Leverhulme Centre for Human Evolutionary Studies, Department of Archaeology and Anthropology, University of Cambridge, Fitzwilliam Street, Cambridge CB2 1QH, UK

**Keywords:** human evolution, modern human origins, African prehistory, dispersals, human evolutionary genetics, Holocene filter

## Abstract

The evolution of modern humans was a complex process, involving major changes in levels of diversity through time. The fossils and stone tools that record the spatial distribution of our species in the past form the backbone of our evolutionary history, and one that allows us to explore the different processes—cultural and biological—that acted to shape the evolution of different populations in the face of major climate change. Those processes created a complex palimpsest of similarities and differences, with outcomes that were at times accelerated by sharp demographic and geographical fluctuations. The result is that the population ancestral to all modern humans did not look or behave like people alive today. This has generated questions regarding the evolution of human universal characters, as well as the nature and timing of major evolutionary events in the history of *Homo sapiens*. The paucity of African fossils remains a serious stumbling block for exploring some of these issues. However, fossil and archaeological discoveries increasingly clarify important aspects of our past, while breakthroughs from genomics and palaeogenomics have revealed aspects of the demography of Late Quaternary Eurasian hominin groups and their interactions, as well as those between foragers and farmers. This paper explores the nature and timing of key moments in the evolution of human diversity, moments in which population collapse followed by differential expansion of groups set the conditions for transitional periods. Five transitions are identified (i) at the *origins* of the species, 240–200 ka; (ii) at the time of the first major *expansions*, 130–100 ka; (iii) during a period of *dispersals*, 70–50 ka; (iv) across a phase of local/regional *structuring* of diversity, 45–25 ka; and (v) during a phase of significant extinction of hunter–gatherer diversity and expansion of particular groups, such as farmers and later societies (the *Holocene Filter*), 15–0 ka.

This article is part of the themed issue ‘Major transitions in human evolution’.

## 1. Introduction

Questions about the origins of modern human diversity, the diversity observed among living people and their unique ancestors, are as old as the discipline of Anthropology. Early evolutionary models sought answers in the regional differences in Pleistocene hominin morphology to account for contemporary differences among human groups [1,2]. These multiregional models suffered from an over-emphasis on differences, positing independent or partially independent deep evolutionary trajectories of human populations in different parts of the world. However, morphological and genetic research since the late 1980s established that all humans are more closely related to each other than to any Late Quaternary hominin group. This showed that humans share an ancestor who lived after other hominin lineages had diverged [3–8], confirmed through palaeogenomic studies [9,10]. These and subsequent studies have also shown that the people of Africa are more diverse than all other human populations [11], and together with the fact that the earliest anatomically modern human fossils are found in Ethiopia [12–14], point to sub-Saharan Africa as the place of origin of the species around 200 000 years ago (200 ka), consistent with genetic results [15].

Nevertheless, the 200 000-year-old history of the species remains only patchily known, with a poorly mapped African origin, and shaped by major geographical and demographic expansions after 130 ka, when modern human populations began to disperse first across Africa and later into Eurasia [16,17]. This critical change in population dynamics from a demographically small and geographically localized lineage to an expansive migratory one is one of the keys to understanding the mechanisms that underpin the evolution of human diversity. The changes in diversity through time are pronounced, but they were not synchronous throughout the world nor do they appear to have had universal triggers. The diversity of the species was formed by the sum of population-level processes, micro-evolutionary in scale and associated with local ecological conditions that engendered a range of economic, social and cultural changes. The latter often had a geographical dimension in the form of either the expansion or contraction of groups, which created new trajectories and opportunities. Whether some or all of these processes had biological consequences to the groups involved remains one of the big questions behind understanding the events that led to the success of the species, as well as of some groups relative to others.

**Figure 1.**
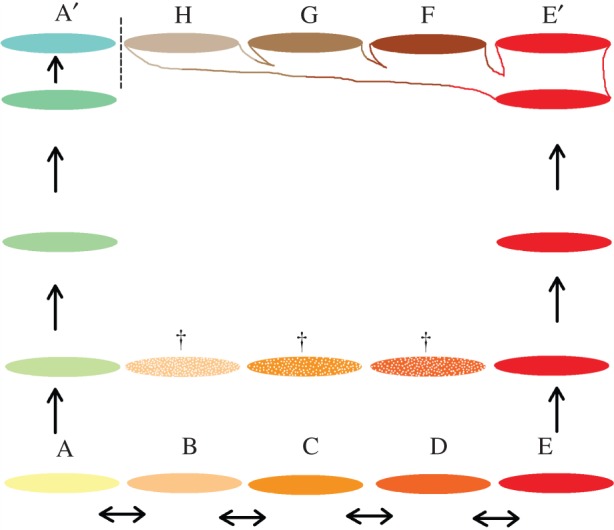
Schematic drawing illustrating the role of extinction of some populations and differential expansion of others in creating population outliers, increasing the distinctiveness of groups and shaping population boundaries.

The combined effects of population growth and population-specific biological change, including the localized admixture with other Late Quaternary hominins, led to the substantial increase in human diversity through the history of the species. Countering that long-term trend, the extinction of many small populations, each of which carried unique characteristics, has removed diversity from the species pool at particular times. The interplay between added and lost variation shaped the distinctiveness of groups through time, strengthening and weakening population boundaries. This is particularly important in the early part of the process, when the deeper structure of human diversity became established (figure 1).

However, any attempt at modelling the history of *Homo sapiens* is constrained by the limited number of African fossils, both before and after the out-of-Africa dispersals. This means that it is impossible at present to contrast the morphology and behaviour of the first humans in Eurasia with that of their African ancestors and immediate relatives, and thus to interpret the processes that shaped human adaptive responses to environmental expansion and/or contraction. Nevertheless, much is known about the non-African part of the human story, and ancient genomics is offering major new insights into changes in population size and relationships of early Eurasian human groups [18–23], including the nature and extent of interactions between expanding modern humans and other Late Quaternary hominins [9,10,15,24,25]. Within Africa, the growing archaeological record of Upper Pleistocene humans in the southern and northern margins of the continent [26–28] is beginning to establish parameters to the distribution of populations through time.

The integration of morphological, archaeological and genetic information offers the fullest picture of the pattern of human diversification, as well as insights into some of the processes involved, such as inter-group admixture. However, in order to test current interpretations of population relationships, as well as help frame questions about the environmental and competitive conditions that underlie significant moments in the history of the species, it is necessary to build a theoretical framework that places major changes in the biology and behaviour of the species, as well as changes in population size, distribution and mobility, in ecological and geographical context. Such changes were continuous, complex and dynamic. However, because climate change was not homogeneous through time, there were periods of greater stress on survivorship and of greater opportunity for population growth. Some of these periods represent transformative moments, shaped by the interplay between the loss of variation through extinction of some populations and the gained variation through the expansion of others. This paper proposes five periods of transition in the evolutionary history of our species, and explores their consequences on levels of diversity.

## 2. Diversity in time and space

Human diversity is defined by the sum of unique biological and cultural variation within our species. *Homo sapiens* has long been characterized as ‘polytypic’ because of the extent of differences among populations. Nevertheless, there has been much debate about the nature and extent of human ‘polytypism’. The rate and degree of differentiation of a population is affected by five factors: (i) the demographic conditions (population size and structure), which determine the potential for drift, both at the point of separation from an ancestral source and through time; (ii) private selective forces resulting from the stress on individual survivorship and fertility in particular environments and not present elsewhere (i.e. selection acting on only one population of the species); (iii) levels of isolation that, by counteracting the homogenizing effects of gene flow, lead to inter-population diversity; (iv) levels of intra-population gene flow that promote the spread of individually acquired novelties; and (v) the time elapsed since separation from an ancestral source. Therefore, what determines the intra- and inter-population levels of diversity within the species is a combination of history, geography and demography—in other words, the evolutionary geography of the species and its populations.

Evolutionary geography is the branch of biology that investigates the role of spatial factors in the evolutionary process [17]. It investigates the environmental and demographic conditions that shaped vicariance and dispersal events in the history of a lineage, and explores the evolutionary causes and consequences of these events. All evolutionary processes have a geographical dimension—climatic and tectonic events that create contingent factors, and allopatry that leads to the accumulation of micro-evolutionary differences, whether among species or among populations within a species, by restricting gene flow and creating different selective landscapes that determine pressures on survival, range and demography [17].

In the case of human evolution in the Pleistocene, when hominins became transcontinental, spatial differences in environmental conditions and the geographical distance between populations became the main contexts for adaptive and neutral evolutionary change. Therefore, in order to explore whether there are particular transformative periods in the evolutionary history of humans, it is critical to understand the relationship between geography and the other two evolutionary dimensions of populations—demography and their duration as distinct entities, from the moment of founding or separation from the ancestral source to their disappearance either by extinction or by reintegration into the ancestral or another population. The backdrop of these relationships is climate change.

The role of climate in evolution has been a matter of debate, from those who see climatic change as a sufficient and necessary cause for evolutionary change [29], to those who emphasize coevolutionary processes as the main drivers of evolution independent of climatic shifts [30], to those who stress the integration of the two, recognizing the role of climate in changing environmental conditions and a population's particular competitive context in shaping its evolutionary response [31–33]. In the case of modern human evolution, climate change triggered moments of environmental expansion and contraction that affected the size and geographical distribution of populations, while the localized ecological conditions of different groups, combined with adaptive novelties that increased carrying capacity, determined the extent to which these demographic and spatial shifts led to major changes in a population's history, including dispersals and extinction.

Although human population size has increased exponentially since the origin of the species, particularly, in the last 10 000 years [34], current evidence suggests that Late Quaternary hominin populations, including humans, experienced significant fluctuations in size [17,35]. During adverse periods, resource depletion, nutritional deficits and increased habitat fragmentation would have repeatedly led to population contraction. Depending on the ecological conditions, such periods of reduced population could be either endured or lead to the local extinction of groups. When the adverse conditions were at a regional level, the local effects would be amplified, and entire populations may have become extinct or differentiated through genetic drift. Conversely, periods of resource abundance would have led to demographic expansions, leading to dispersals and the expansion of the population's range, new adaptive environments and potential inter-group conflict [36]. While conditions were probably sufficiently dynamic to continuously affect population sizes at a local scale, it is periods of rapid shift from adverse to favourable conditions that have the greatest impact on diversity.

Several factors promote the differential success of populations following a period of contraction—the same climatic change will cause very different patterns of environmental deterioration across regions, leading to localized areas of endemism (refugia) and depopulation, the latter offering niche opportunities to the former upon recovery. Furthermore, environmental recovery is not simultaneous at regional and continental scales, so even among groups who survive periods of adversity, population expansion will begin among some before others. Finally, different ecological circumstances promote different adaptive strategies, whether biological or cultural, some of which can make a population's capacity for resource exploitation greater than others. Thus, the particular interactive effects between population collapse and expansion following a rapid shift from environmental adversity to recovery set the conditions for the periods of transition in the evolutionary history of modern humans.

I have identified five phases in which extinction and contraction are followed by significant differential expansion, and thus set the conditions for a shift in human diversity, the basis for an evolutionary transition—(i) at the *origins* of the species, 240–200 ka; (ii) at the time of the first major *expansions*, 130–100 ka; (iii) during a period of major *dispersals*, 70–50 ka; (iv) across a phase of multiple extinctions/expansions of local/regional expression leading to the *structuring* of diversity, 45–25 ka; and (v) during a phase of significant extinction of hunter–gatherer diversity and expansion of particular groups, such as farmers and later societies (the *Holocene Filter*), 15–0 ka.

## 3. The evolutionary history of *Homo sapiens*

Before discussing such transitions in the evolutionary history of *H. sapiens*, when populations changed, dispersed or became extinct at a greater rate than others, it is necessary to consider the constraints that limit their identification. Any attempt at establishing the pattern and process of the evolution of human diversity is hampered by the incompleteness and complexity of the information, and as the fossil record increases and more ancient genomes are described, the transitions described here will become more precise and probably more numerous. Nevertheless, identifying these transitions is a critical step in developing a comprehensive model for the evolution of human diversity.

### (a) *Origins*, 240–200 ka, MIS 7

The early history of *H. sapiens* remains largely unknown. African Middle Pleistocene hominins share morphological and archaeological characteristics with European remains [37,38], reflecting an early/mid Middle Pleistocene dispersal event across the two continents, which probably originated in Africa [39]. This shared Afro-Eurasian Middle Pleistocene ancestry is supported by palaeogenomic data on the descendant species—humans, Neanderthals and Denisovans [25]. Subsequent evolutionary changes within Africa are poorly documented. Fossils from two Ethiopian sites, Omo Kibish and Herto, place a modern human morphology between 195 ka and 160 ka [13,14,40,41], from the end of marine isotope stage (MIS) 7 to MIS 6. These fossils show unique universal features, such as human cranial proportions and a chin [8,12], but are also substantially different from each other and from more recent humans. The lack of a more comprehensive sample of Middle Pleistocene African hominins precludes establishing whether these differences reflect the standing variation in the ancestral population or private local trajectories. The diversity they reveal may be extended with the inclusion of the MIS 6 fossils from Djebel Irhoud, Morocco, within this early *sapiens* hypodigm [42,43], and even further by that of Ngaloba, Tanzania [44,45]. On the basis of the Ethiopian discoveries, East Africa has been suggested as the area of origin for the species, consistent with some genetic studies [46], and supported by models of the role of refugial networks during climatic instability [47,48]. However, other genomic analyses point to a complex spatial pattern for the origins of modern humans [49], while a case for a southern African origin has been made on ecological [50], archaeological [51] and genetic grounds [52], although the latter is strongly influenced by the current distribution of the few surviving African hunter–gatherer populations [53].

**Figure 2.**
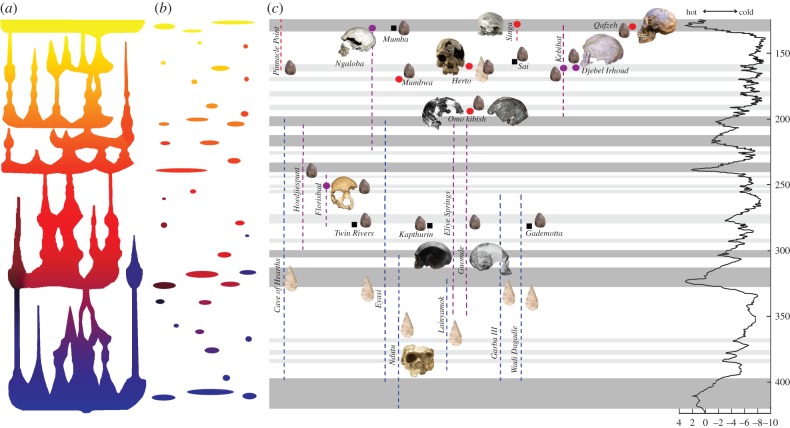
(*a*) Cartoon of the complex phylogenetic pattern through time, shaped by repeated expansions, extinctions and assimilations; (*b*) geographical expression of a complex phylogenetic pattern in the fossil and archaeological records; (*c*) distribution of key fossil specimens and archaeological sites in Africa between 400 and 130 ka in the context of climate change. In (*c*), blue lines represent fossils of *Homo heidelbergensis*, purple lines and circles fossils of *Homo helmei* and red lines and circles fossils of *Homo sapiens*. Archaeological sites are represented by black squares, Acheulean industries by a hand-axe, and Middle Stone Age industries by a Levallois point.

The archaeological record of modern human origins is complex. All early modern human fossils have been found in association with Middle Stone Age (MSA) lithic traditions, but the MSA pre-dates the earliest known fossils of *H. sapiens* [53]. Furthermore, the modern human fossils of Herto were found in the context of an archaeological industry that has both MSA and Acheulean elements [54], while at the similarly dated site of Pinnacle Point in South Africa, behavioural innovations interpreted as reflecting the more complex cognition of modern humans are observed [51]. The complexities of the archaeological and (scant) fossil record of the period 300–130 ka in Africa suggest a prolonged phase in which repeated expansions and contractions would have led to the recurrent assimilation within a single lineage of biological and behavioural novelties acquired locally during periods of allopatry (figure 2).

A date for the origins of the modern human lineage at the end of MIS 7 (*ca* 200 ka) is consistent with genetic estimates of the last common ancestor of living humans based on mitochondrial DNA (mtDNA) [15], but slightly older than the estimates obtained from other genetic data (150–100 ka [55]). Although genetic coalescence dates have large margins of error, these estimates suggest that the *sapiens* morphological clade, defined from the point when modern human uniquely derived traits first appear in the fossil record, pre-dates the last common genetic ancestor of living people. Thus, in the absence of a fuller late Middle Pleistocene African fossil and archaeological record, the spatial and demographic parameters of early modern human evolution in Africa remain unclear. This is critical, as the geographical location and ecological context of the ancestral population shaped the potential for drift and the selective environment under which a modern human phenotype evolved [53,56], and thus set the adaptive characteristics shared by all humans, and which were later exported through dispersals across the world.

### (b) *Expansion*, 130–100 ka, MIS 5

The period between 128 and 72 ka corresponds to a long and complex interglacial (MIS 5) [57], which began by a climatic shift from hyper-glacial to warm conditions, accompanied by a period of major precipitation in the tropics, and high lake-levels, expansion of forests and greening of deserts [58]. This period is the backdrop of the first large-scale expansion within the time-depth of living human ancestry. Modern human fossils are found across Africa and neighbouring Western Asia—the fossils of Singa (Sudan, 133 ka [59,60]), Mumba (Tanzania, 130–109 ka [61,62]), Aduma (Ethiopia, 105–79 ka [63,64]), Garba III (Ethiopia [65]), Klasies River Mouth (South Africa, 115–60 ka [66,67]) and in the Levant, the famous fossils from Skhul and Qafzeh [68,69]. Several other human fossils have dates that fall within MIS 5/MIS 4, such as Mugharet el'Aliya, Dar-es Soltan 2, El Harhoura 1, Témara, Haua Fteah and Taramsa Hill in North Africa [42,70–74], Diré-Dawa in Ethiopia [75] and Border Cave, Blombos, Die Kelders Cave and Equus Cave in South Africa [76–80]. Although few of these remains are sufficiently complete to allow in-depth comparative studies, together with the changes in the archaeological record of this period, they are strong evidence for the widespread temporal and spatial distribution of modern humans in Africa by this time [53]. Genetic analyses suggest that some contemporaneous African hunter–gatherer groups trace their population history to the early part of the MIS 5 pan-African expansion [15,81].

Besides the clear change in population size evidenced by the number and distribution of archaeological sites throughout Africa, this phase of modern human evolutionary history (from the beginning of MIS 5 (128 ka) to the end of MIS 4 (60 ka)), represents a moment of important behavioural innovations—the first evidence for personal ornaments [78,82–86], for the non-functional use of pigments [87,88], artistic/geometric markings on soft stones [78,89], for the decoration of objects [90], for the use of carefully chosen plants for bedding [91], greater technological complexity, such as the extent of use of bone as raw material, tangs on lithic points, or the development of true microliths and composite tools, with complex mastics and design [26–28,78,92–94], and economic diversification [51]. Other evidence of increasing material expression of social norms and shared symbolic and aesthetic meaning is in the form of burials with objects (such as Qafzeh 11) [95]. These behavioural novelties encompass both technological and socio-cultural changes, and reflect the establishment of regional cultural traditions, some of which may have been of limited duration [96,97]. Such a process of regionalization is consistent with the complex climatic changes that took place during the second half of MIS 5 and MIS 4, including the ‘megadrought’ that affected the tropics 80–70 ka [98].

The recent discovery of modern human teeth dated to approximately 80 ka in China [99] indicates that the early MIS 5 pan-African human expansion reached beyond western Asia, and established the first modern human populations in eastern, and presumably southern/southeastern Asia [100] prior to the main dispersal events that took place 20 000–30 000 years later. This interpretation is supported by recent genomic evidence that shows that the Altai Neanderthals carry the signature of admixture with a modern human population that dispersed into Eurasia before the ancestors of living non-African peoples sampled to date [25], a population which has presumably become extinct since it has not left a genetic signature among living Eurasians. The question of why this first modern human expansion into Eurasia did not succeed has been asked since it was shown that the modern human Skhul/Qafzeh population was replaced by Neanderthals in the Levant. The new palaeontological and palaeogenetic discoveries throw light on two aspects of this issue. They suggest that modern humans reached Eurasia approximately 130 ka not just as part of the ecological extension of African environments, but as a part of a true dispersal event that gave rise to populations as far as eastern Asia. Since there are no apparent technological underpinnings to this expansion, the underlying success of this group must be sought in other aspects of its behaviour and biology. However, this success was not sustained. It is here that the palaeogenomic evidence provides unique insights—the Altai Neanderthal is the only case so far in which the children from a human-Neanderthal sexual encounter were brought up as Neanderthals, suggesting that, by that stage, the populations of early MIS 5 Eurasian modern humans were small and outcompeted.

### (c) *Dispersals*, 70–50 ka, MIS 4–3

A very large proportion of living people, both in Africa and outside the continent, share a common ancestor estimated to have lived at the end of MIS 5 or during the early phases of the subsequent glacial phase, MIS 4. Genetically, this ancestor is identified as the population that gave rise to mtDNA haplogroup L3 and descendant lineages, today found across most of Africa and throughout the rest of the world [15,101]. In the absence of palaeogenomic data for Pleistocene African fossils, it is not possible to locate this population geographically. However, the pattern of distribution of genetic descendants throws light on its possible geography and history. Two main groups of contemporary people trace their matrilineal ancestry to this population—a significant proportion of contemporary Africans (approx. 70% of North Africans, 50% of East Africans, 40% of West Africans, 25% of both Central and Southeast Africans, and approximately 10% of South Africans [102]), and the rest of the world [81]. Whether Eurasian populations dispersed in one or multiple events remains a matter of debate. The morphological and archaeological differences between Australo-Melanesians and other Eurasian peoples have been explained as reflecting separate dispersals from a structured African population during MIS 5 [16]. However, genetic results suggest that the observed differences may have arisen from sequential dispersals from a single ancestral source [22], enhanced by differential admixture with archaic hominins [103,104].

Taken together, this evidence indicates that, by the second half of MIS 5, (i) African populations were structured into different groups, consistent with the prehistoric evidence for regional differentiation and deteriorating climatic conditions; (ii) that one of these groups (‘X’) underwent sequential waves of expansion that led first to the establishment of descendant populations within Africa, creating the north–south cline of matrilineal relatedness in this group; (iii) that one of the descendant groups expanded out-of-Africa (‘X-ooA’), came into contact with Neanderthals, and assimilated some Neanderthal individuals into the group [103,105]; and (iv) that this population carrying Neanderthal admixture eventually gave rise to two or more dispersals across Eurasia (between ?60–50 ka), leading to further private admixture events with both Neanderthals and Denisovan hominins along the different routes of expansion [104] and establishment of the core population structure outside Africa. If this sequence is correct, the most parsimonious location for the ‘X’ ancestor would be northeast Africa. The location of ‘X-ooA’ remains unknown—the northern Nile Valley, southern Levant and Arabia have all been suggested as potential localities, all of which would be consistent with range expansions into Neanderthal territory and the opportunity for the admixture event with Neanderthals shared by all Eurasians to take place.

The pattern of population dispersals in Eurasia between 70 and 50 ka is being revealed by ancient genomics. A series of studies have shown that non-African human diversity has its genetic roots in the early splitting of groups from the ‘X-ooA’ ancestral population, leading to the establishment of a population ancestral to Australo-Papuans, one ancestral to the people of Western Asia, and one ancestral to all other groups, which divided relatively quickly into one population that gave rise to Western Eurasians and another to Eastern Eurasians [19,20,22,23]. The genomes of fossils such as Ust’ Ishim, in Russia [18], indicate that other groups formed at this time who left no living descendants. However, the geographical expression of this process is unclear at the moment. The fact that the above groups acquired a genetic identity at this time points to sufficient allopatry for inter-group gene flow to be restricted. Nevertheless, the archaeological record shows, consistently across multiple regions, that the Eurasian regional structure that persists to the present was only established at the end of this period.

What is known about African populations at this time suggests different regional trajectories, consistent with observed environmental differences [98]. In southern Africa, the number of archaeological sites and complexity of the material culture between 75 and 60 ka greatly increases to form a series of regional cultural phenomena that last until the end of the period [77,78,89–94]. The reasons for their disappearance, and its demographic implications, remain a matter of debate [78]. The re-excavation and dating of the Haua Fteah, Libya, have securely placed modern humans in Cyrenaica at this time [73]. For eastern African populations, the period 70–50 ka should be one of population recovery after the megadrought; unfortunately, the number of dated archaeological sites is too small to interpret the data at population level. Nevertheless, two sites (Porc Epic in Ethiopia, and Mumba in Tanzania) record the first elements of the African Later Stone Age (LSA) at this time [62,106], somewhat earlier than in South Africa [107], but the significance of this process in relation to the evolution of African diversity remains unclear.

### (d) *Structuring* of existing variation, 45–25 ka, MIS 3–2

The evolutionary history of human populations in Eurasia between 50 and 15 ka is relatively well known. The record shows that between 55 and 45 ka, modern human populations were present in western Asia [108], eastern Europe [24], southeast Asia [109,110] and Australia [111], consistent with the population dispersals that took place in the preceding phase. Timing the first modern humans in central and western Europe is complex and inter-twined with the contraction of Neanderthals and changes in their behaviour. The first lithic industry generally accepted as reflecting modern humans, the Aurignacian, is recorded in Europe between 45 and 43 ka at sites such as Willensdorf [112], although earlier, potentially non-lasting, occupations may have taken place [113,114]. A similar date of approximately 40 ka marks the first modern human fossils in East Asia (Tianyuan [115]), slightly older than the earliest dated microlithic industries of India [116] and Sri Lanka, where the Batadomba-lena and Fa Hien caves show the exploitation of rainforests at this time [117]. However, the complexities of the South Asian Upper Pleistocene population history, its rare hominin fossils despite a rich prehistoric record in the sub-continent, as well as the critical biogeographical role of the region in dispersals from western to southeast Asia, suggest that the first modern populations there are yet to be found [118].

This is the critical period that shaped non-African diversity, when local patterns of extinction/survivorship magnified the differences acquired during the process of population dispersals of the preceding phase and created the basal inter-population structure among non-Africans, magnifying the differences acquired during the process of population splitting in the preceding phase. Besides the signature of an ancestral admixture event with Neanderthals [9,10], some of these non-African groups show evidence of population-specific localized admixture events with archaic hominin individuals, both Neanderthals [18,24] and Denisovans [103–105], which further magnified the structured-population differences.

The archaeological record of the last glacial period in Africa shows the major change from the MSA to the LSA throughout the continent. Only a handful of fossils, widely separated geographically and temporally, are known from this time [58]. Among these, a 36.2 ka South African fossil shows no close relationship to recent African groups, but instead a pattern that allies it to similarly aged material from Eurasia [119].

The Last Glacial Maximum (LGM, 26.5–19 ka [120]) was a global event, but the consequences for populations across the world differed [121]. Northern latitudes were partly covered by glaciers or developed polar desert conditions and became depopulated; the tropical desert and semi-desert belts became hyper arid, further reducing land availability and shifting animal distributions; in between extremes of cold and aridity, populations contracted into refugia. In some cases, populations responded by major technological innovations, such as the first recorded manufacture of pottery at the caves of Yuchanyan and Xianrendong in China [122,123], produced by mobile hunter–gatherers during the LGM. The particulars of regional patterns of population collapse during the LGM are beyond the scope of this paper, but where prehistoric records exist, the evidence suggests that all regional populations appear to have been affected, representing a moment of species-wide potentially significant loss of diversity.

### (e) *The Holocene Filter*, 15–0 ka, MIS 2–1

The last deglaciation was a staggered process. The melting of the northern hemisphere glaciers, caused by an increase in northern summer insolation 20–19 ka, resulted in a rapid rise in sea-level; this was followed by the onset of deglaciation of the West Antarctic Ice Sheet between 15 and 14 ka [120], causing an abrupt further rise in sea level, and leading to the onset of the African Humid Period and interglacial conditions worldwide. In terms of human population history, the last 15 000 years represent the period in which local population adaptive trajectories become regional, and in some cases continental, changing the distribution and structure of human diversity throughout the world. This process was mediated by the concatenated consequences of demographic and economic change, leading to the unprecedented differential expansion of some groups in relation to others.

The first phase of the process corresponds to the expansion of some hunter–gatherer–fishers from localized refugia across vast spaces newly created by abrupt climate change. In Africa, the forests expanded, the southern Cape coastline contracted and an extensive hunting–fishing population and/or trading network spread from the northern Kenyan lakes to the Nile, across the Sahel and into the greening Sahara [124,125]. Across western and northern Eurasia, hunter–gatherers expanded north [126], recolonizing the vast territory previously covered by ice and permafrost, a process that changed the genetic landscape from Europe to Siberia [20,127], and also contributed to the peopling of the Americas [21,128]. Knowledge of this period in west, south and east Asia is very variable, but each region sees the internal expansion of foraging societies from local areas of endemism. Southeast Asia is a unique case, since the Late Pleistocene/Early Holocene is not a period of niche expansion but reduction associated with the flooding of the Sunda landmass. Finally, rising sea-levels also broke-up the landmass of Sahul, although archaeological, fossil, linguistic and genetic evidence suggests that Aboriginal Australian and Papuan populations already had differentiated trajectories [129].

This massive expansion of hunter–gatherers led, for the first time, to the occupation of all regions and environments of the world with the exception of Polynesia and Micronesia. In west, south and east Asia, where these expansions occurred within relatively bounded areas, population density and distribution affected mobility, leading to economically intensive foraging strategies that included cereal processing, fishing and semi-sedentism, and eventually farming. Hunting and gathering as a mode of subsistence was almost entirely replaced throughout the world in the course of the last 10 000 years. Economically, hunter–gatherers followed several different strategies, including different levels of reliance on big-game versus small-prey hunting, forest animal produce (including bee-keeping and bird-catching), fishing and shell-fishing, key plant produce (such as particularly nutritious nuts) and levels of storage, with increasing evidence for the use of fired clay pots in hunter–gatherer archaeological contexts [36,122,123]. However diverse their economies, the demographic parameters associated with hunter–gatherer lifestyles have some relatively homogeneous aspects [130]—for most of them, the dispersed nature of the resources and their seasonal/random unreliability meant that foraging had to take place in small social units (often kin-based), and that the areas needed for foraging were relatively large. Therefore, for the most part, hunter–gatherers were spatially structured and lived at low population densities, with likely high inter-group genetic diversity.

Farming and animal husbandry removed one of the biggest dangers of a hunter–gatherer lifestyle—its unpredictability. By relying on resources that could be controlled in terms of amount, reproduction and storage, they reduced the threat of famine and the need for large foraging territories. The development of carbohydrate-rich and dairying diets allowed for early and reliable weaning of infants, and various studies show that traditional farmers have lower infant and child mortality than hunter–gatherers today [130]. The inevitable agricultural population expansion led to the progressive need for ever more farming land, and direct competition for it with both wildlife and foragers. Slowing down this process was the farmers' need for protein and nutritious foods, which they obtained by trading with hunter–gatherers at the edge of forests, mountains and lakes. The latter provided meat, honey, fish and key plants and these trading relations commonly became formalized in various ways. When this process took place, many hunter–gatherers abandoned their original language to adopt that of their trading partners and settled near/within farming villages [131].

The extent to which expanding farmers replaced or assimilated hunter–gatherers has been a matter of dispute for decades [132,133]. The process has been most comprehensively studied in western Eurasia. Archaeological and genetic studies show that early European farmers were migrants from western Asia [134,135], where agriculture first developed [136], and although the genetic impact of their expansion across Europe was very substantial, the proportion of surviving indigenous European hunter–gatherer genes varied through time and space [137–140]. However, subsequent historical movements from the east, particularly during the Bronze Age, had a further impact on the genetic landscape of Europe [141,142], some of which may have been mediated by infectious diseases [143]. In Africa, the few Late Glacial/Early Holocene human remains show morphological traits that are not common today [144–146]. Together with the correspondence between genetics and the distribution of the Bantu languages of farmers [11] and disappearance of hunter–gatherers across most of sub-Saharan Africa, this suggest a similar process of population replacement with partial assimilation through which hunter–gatherer, as well as some very rare ancestral variation, survived [147]. Thus, farming and later population movements had a massive impact on the distribution and diversity of human populations in most regions of the world [133]. There are exceptions in this scenario—most notably parts of southern Africa and Australia, where a hunting and gathering lifestyle continued.

Although the biological consequences of Holocene population movements and expansions are complex and reticulated, the impact of this process on the cultural landscape today is profound [148]. Languages give us a good measure of this: 347 (or approx. 5%) of the world's languages have at least 1 million speakers and account for 94% of the world's population; the remaining 95% of languages (approx. 6500) are spoken by only 6% of the world's people [149]. Human variation today is greater than at any point in the history of the species, an inevitable consequence of being more than 7 billion individuals. However, the size of the human population has not increased continuously, either spatially or through time, and the repeated differential survivorship and expansion of some groups and not others has led to the progressive loss of portions of ancestral diversity and the formation of outliers that retain variation that is ‘rare’ in comparison.

## 4. The five transitions in the evolutionary history of *Homo sapiens*

**Figure 3.**
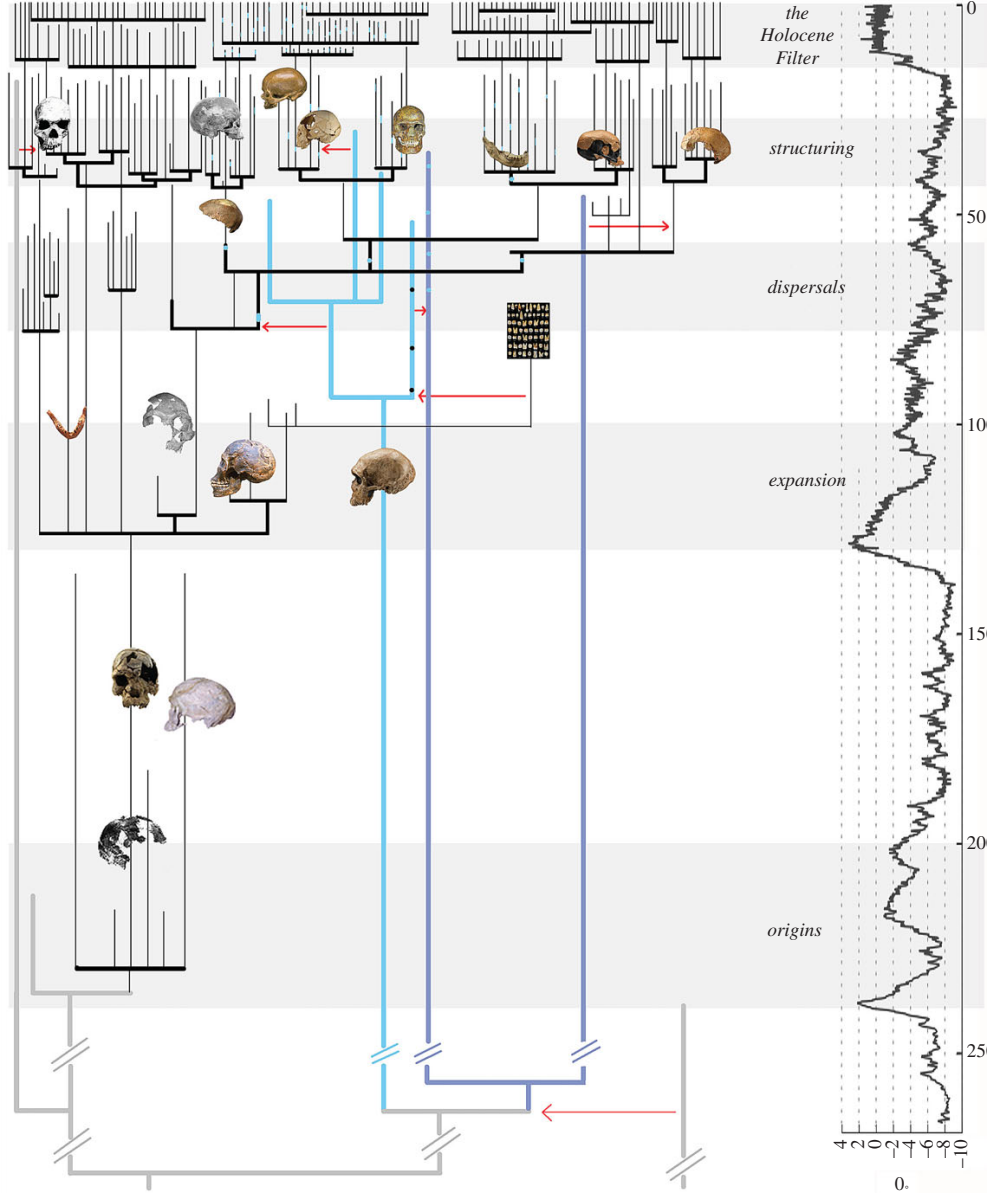
Schematic of the five transitions in the evolutionary history of *Homo sapiens*. Dark blue lines represent Denisovans, light blue lines Neanderthals and grey/black lines modern humans. Red arrows represent events of interspecific admixture. To the right, climate variation (*x* axis) based on oxygen isotope data through time (*y* axis).

The diversification of modern humans was, necessarily, a gradual and continuous process. However, as briefly outlined above, the rate at which population change took place was different across space and, especially, across time. Major changes in the biology and/or behaviour of human populations follow from periods of intense selection and/or drift, when pressure on survivorship was a major challenge, or from periods of rapid population growth, when biological and cultural novelties accompany demographic expansion. In evolutionary terms, transitions often conflate both mechanisms. The biological and/or behavioural changes that take place during population collapse are only revealed if the group survives, and particularly if it subsequently expands demographically, and thus magnifies the differences acquired during the period of increased drift, to which the novel variation due to population growth is added. However, not all populations survive severe bottlenecks, or recover from one at the same rate. In the first case, when small groups become extinct, the areas left vacant fuel the pace of expansion of neighbouring groups when conditions improve; in the second case, when demographic recovery is slow, the scope of success depends on the distance from the wave of expansion of others. The result is that periods of rapid population growth of some groups are also associated with the extinction of others, acting like a filter. Therefore, transitions result from the interaction between (i) the addition of variation during population collapse and rapid expansion and (ii) the loss of variation due to localized population extinction and/or their partial assimilation into expanding groups. These transitions are thus the moments when the level, distribution and structure of diversity in the history of modern humans changed significantly (figure 3).

Using the evidence outlined above, I have identified five such transitions in the history of modern humans:

(1) *Origins:* 240–200 ka (MIS 7), Africa. This transition involves the evolution of the distinct, universal morphological traits of *H. sapiens*, and characterises the change from archaic to modern morphology in one African lineage. The size of Africa, together with the paucity of fossils and securely dated archaeological sites, precludes establishing patterns of population extinction/assimilation at this time. Thus, the chronological and spatial framework for the disappearance of ‘archaic’ African populations is unknown, although the late survivorship of non-current combinations of morphological traits and rare ancient genetic lineages, supported by the apparent ‘ragged’ disappearance of MSA technologies, suggests modern human hegemony in Africa is relatively recent. With only a handful of fossil crania between 200 and 12 ka [150], and no Pleistocene ancient genomes, putting a date to the last African ‘archaic’ is not possible. However, a working hypothesis would place their disappearance at a similar time as in Eurasia, at the beginning of the *Structuring* phase, when a combination of human population growth, shown by the number of LSA sites across the continent, and climate adversity may have led to their final extinction.
(2) *Expansion:* 130–100 ka (MIS 5), Africa, with Eurasian incursion. This transition follows a major glacial period, during which hominin and human African populations would have become fragmented and diversified through drift and adaptation. Little is known about this process, but the subsequent modern human expansion is shaped by a strong shift in demography and behaviour, and leads to the establishment of discrete regional populations of *H. sapiens* across southern, eastern and northern Africa, the probable assimilation of individuals from archaic African groups in these regions, as well as the temporary occupation by humans of the Levant, possibly Arabia and south Asia, with descendant populations as far as China. The outcome of this transition was the structuring of African populations, including the population ancestral to a large portion of Africans and all non-African groups.
(3) *Dispersals:* 70–50 ka (MIS 4–3), Africa, Eurasia and Oceania. In eastern Africa, this period follows the end of a megadrought, when populations would have expanded as ecological conditions improved. One of these populations would have given rise to the Eurasian dispersals that characterise this period. In both southern and northwest Africa, local populations flourish until approximately 60 ka, when a period of decline is suggested by the archaeological record, while in east Africa, this phase marks the origins of the LSA. Out of Africa, this phase takes the form of an adaptive radiation, with a major expansion of populations that, by the end of the period, have colonized vast areas of Asia and reached the Sahul landmass. This critical period is extremely poorly represented in the fossil record of the species, and most of what we know about it in Eurasia derives from genetic and genomic inferences.
(4) *Structuring:* 45–25 kyr (MIS 3–2), Africa, Eurasia and Oceania. This is the phase in which the conditions of the preceding expansions end, some populations become extinct, others progressively fragment, and inter-group diversity increases. The main structure of living human diversity is established during this phase, with the formation of regional patterns that have been ‘re-shuffled’ since. It also corresponds with the period of extinction of Eurasian archaic populations.
(5) *The Holocene Filter:* the last 15 000 years (MIS 2–1), global. This transition is the outcome of a sequence of events, from the extinction of populations during the LGM, to the subsequent differential and asynchronous expansion of hunter–gatherers, farmers and more recent groups creating a layered process in which the species diversity changed through the loss of inter-group diversity (some dating to the origins of the lineage) and the gain of diversity by large population growth.

## 5. The importance of mapping the evolution of human diversity in terms of its major transitions

Given the paucity of information about the Late Quaternary African record, as well as the complex spatial and demographic patterns of global human diversification, the identification of key periods in which the level and distribution of human diversity changed significantly provides a working model for exploring the climatic, ecological and demographic factors that shaped human biological and cultural adaptation. Some of these adaptations are global, and must lie at the root of human resilience and success; others are continental and reflect the features that promoted major geographical expansions into novel territories, while yet others are recent and regional, and reflect local change, migration and assimilation of indigenous groups. Together they define a hominin species that is phylogenetically, spatially and demographically unique.
